# Effects of the periocular botulinum toxin on the ocular surface and anterior chamber: a prospective study in patients with hemifacial spasm and blepharospasm

**DOI:** 10.1007/s10792-023-02672-8

**Published:** 2023-04-26

**Authors:** María Dolores Romero-Caballero, Juan Antonio Miralles de Imperial-Ollero, Elena Sarabia-Marín, María Paz Villegas-Pérez

**Affiliations:** 1 Ophthalmology Service, Hospital General Universitario Reina Sofía, Avenida Intendente Jorge Palacios, 1, 30003 Murcia, Spain; 2 Department of Ophthalmology, Faculty of Medicine, Instituto Murciano de Investigación Biosanitaria (IMIB) Virgen de la Arrixaca, University of Murcia, 30120 Murcia, Spain

**Keywords:** Acute angle-closure, Botulinum toxin, Facial dystonia, Pentacam

## Abstract

**Purpose:**

To analyze using Pentacam^®^, the corneal and anterior chamber changes following periocular botulinum toxin injection in patients with facial dystonia.

**Methods:**

Prospective study that included patients with facial dystonia that were going to receive a periocular botulinum toxin injection for the first time or six months or more after the previous injection. A Pentacam^®^ examination was carried out in all patients before and 4 weeks after the injection.

**Results:**

Thirty-one eyes were included. Twenty-two had a diagnosis of blepharospasm and nine of hemifacial spasm. Analysis of corneal and anterior chamber parameters revealed a significant decrease in iridocorneal angle after botulinum toxin injection (from 35 ± 10º to 33.8 ± 9.7º, *p *= 0.022). No other corneal or anterior chamber parameters changed significantly after the injection.

**Conclusions:**

Periocular botulinum toxin injection causes narrowing of the iridocorneal angle.

## Introduction

Botulinum toxin is a neurotoxin, a highly specific potent neuromuscular inhibitor that produces temporary chemical denervation by blocking the release of acetylcholine in the motor plaque [1, 2]. Previous studies have documented that the effect of the neurotoxin begins a few days after the injection, peaks at 15–20 days disappears progressively at 3–5 months [2].

Botulinum toxin is currently the main treatment for some ophthalmological diseases, such as some types of strabismus [2, 3] and facial dystonia. Facial dystonia comprises various diseases, such as essential blepharospasm or hemifacial spasm [4–7], characterized by a disproportionate tonic contraction of the orbicularis oculi that causes involuntary eyelid closure. In severe cases, this dystonia may impact the patient’s quality of life and even result in functional blindness [8–10].

Facial dystonias may effectively be treated with periocular subcutaneous injection of botulinum toxin [5, 11, 12]. These injections produce few local complications, such as ecchymosis, ptosis, or diplopia, and do not generally have systemic effects [13]. However, other angle-closure glaucoma attacks [14–17] have been reported after periocular botulinum toxin injection, although the periocular injection of this drug has also been proposed for the relief of pain in acute angle-closure glaucoma [18]

The purpose of this study was to assess using Pentacam^®^ the effects of periocular botulinum toxin injection on the ocular surface and anterior chamber parameters in patients diagnosed with essential blepharospasm and/or hemifacial spasm. We document for the first time a narrowing of the anterior chamber angle after the injection that may be the cause of the angle-closure glaucomas observed after these injections.

## Methods

This is an observational prospective study. The research protocol followed the guidelines of the Helsinki Declaration and was approved by the Clinical Ethics Committee of our Hospital.

### Inclusion and exclusion criteria

We included patients aged > 18 years with the diagnosis of hemifacial spasm and/or essential blepharospasm that were going to receive treatment with periocular botulinum toxin A. The patients included were either naïve when this was their first treatment with botulinum toxin or patients that had received previous doses but not in the previous 6 months.

The exclusion criteria were contact lens use in the previous 4 weeks and refractive, eyelid, or ocular surgery in the previous 6 months.

### Ophthalmic examination

All the patients had a complete ophthalmic examination, that included best corrected visual acuity (BCVA), slit lamp biomicroscopy, and anterior segment Pentacam^®^ examination one day before and 4 weeks after periocular botulinum toxin A injection.

### Pentacam examination

Anterior segment examination was carried out using Pentacam^®^ (HR, Oculus Optikgeräte GmbH, Germany) which uses a Scheimpflug camera to obtain images of the cornea and anterior segment of the eye. The examinations were performed in the automatic mode by the same experienced examiner under the same lighting conditions. Some patients required the use of a blepharostat for the examination. The automatic protocol used obtains 25 Scheimpflug images in 2 seconds. At least 3 examinations were performed for each eye and the quality of the image (QI) was used to select the highest-quality topography for the study.

From the Pentacam^®^ examination, the following parameters were obtained at baseline and after treatment: i) From the anterior surface (AS) and posterior surface (PS) of the cornea, two keratometric data were obtained: the flattest (K_1_) and the steepest (K_2_) corneal meridian in diopters (D) and mean curvature or simulated keratometry (Sim-k), ii) the central corneal thickness, iii) the anterior chamber depth, volume, and iridocorneal angle. The measurement of the iridocorneal angle was performed automatically by Pentacam^®^ and is the average value of the angle formed between the tangent line to the trabecular meshwork and the line corresponding to the periphery of the iris in the 25 high quality Scheimpflug images performed by Pentacam.

### Technique of botulinum toxin injection

Botulinum toxin (Botox, Allergan Corp., Irvine, CA) was prepared as indicated by the manufacturer (5 UI in 0.1 ml) and injected by the same specialist in the orbicularis muscle, 4 points of the eyelids, two in the upper eyelid and two in the lower eyelid of both eyes, and in additional points also into the corrugator supercilii and procerus muscles, with a 30G needle. The patients with hemifacial spasms, received also injections into the affected facial muscles (zygomaticus major, orbicularis oris, etc.) as required.

### Statistical analysis

Statistical analysis was performed using Stata software version 14 (StataCorp. 2015. Stata Statistical Software: Release 14. College Station, TX: StataCorp LP.). Normality was examined using the Shapiro–Wilk test. To compare the data before and after the treatment, we used the t-Student for paired data if normality was documented, and Wilcoxon signed-rank test for paired data when data were not normal. Differences were considered statistically significant when *p *< 0.05.

## Results

Thirty-one eyes from 20 patients were included. Twenty-two eyes had blepharospasm and 9 eyes had a hemifacial spasm. Fifteen patients (75%) were female and five patients (15%) were male. The mean age of the patients was 54.8 ± 5.8 (mean ± SD). The mean BCVA was 0.7 ± 0.1 LogMAR. After toxin injection, none of the patients had ocular complications such as dry eye, ocular or periocular pain, or eyelid ptosis. Table 1 summarizes the parameters of the Pentacam® examination.

**Table 1:** Corneal topography parameters before and after treatment

	Before toxin injection (baseline)(*n *= 31 )	One month after toxin injection (*n *= 31)	Statistical significance
K1-AS (D)	44.2D ± 2.3	44.4 ± 2.2	*p *= 0.29
K2-AS (D)	45.3 ± 2.1	45.1 ± 1.8	*p *= 0.46
Sim-K AS(D)	44.2 ± 2.3	44.7 ± 1.8	*p *= 0.623
K1- PS (D)	− 6.4 ± 0.5	− 6.4 ± 0.4	*p *= 0.29
K2- PS (D)	− 6.7 ± 0.3	− 6.7 ± 0.3	*p *= 0.46
Sim-K PS(D)	− 6.5 ± 0.4	− 6.5 ± 0.4	*p *= 0.724
Pachymetry (µm)	561.0 ± 30.4	565.1 ± 28.3	*p *= 0.098
AC Volume (mm^3^)	130.9 ± 53.1	130.3 ± 56.4	*p *= 0.84
AC Depth (mm)	2.9 ± 0.6	2.9 ± 0.6	*p *= 0.122
Iridocorneal angle (degrees)	35 ± 10	33.8 ± 9.7	*p *= 0.022*

*AS* Anterior surface, *PS* Posterior surface, *D* Diopters, and *AC* Anterior chamber

*p < 0.05

### Keratometric parameters

The K_1_ and K_2_ values of the AS were 44.2 ± 2.3 D and 45,3 ± 2,1 D, respectively, before botulinum toxin injection (Table 1). The K_1_ and K_2_ values of the PS were − 6.4 ± 0.5 D and − 6,7 ± 0.3 D, respectively, at baseline. The post-injection K1 and K2 values for of the AS and the PS of the cornea can be observed in table no significant differences in the K_1_ or K_2_ keratometry parameters were observed after botulinum toxin injection (*p* = *0.*296).

### Central corneal thickness

Mean central corneal thickness was 561 ± 30.4 μm at baseline and 565.1 ± 28.3 μm one month after injection. Although we observed an increase in corneal thickness after the injection, this difference was not statistically significant (*p* = *0.09*).

### Anterior chamber parameters

**Fig. 1:**
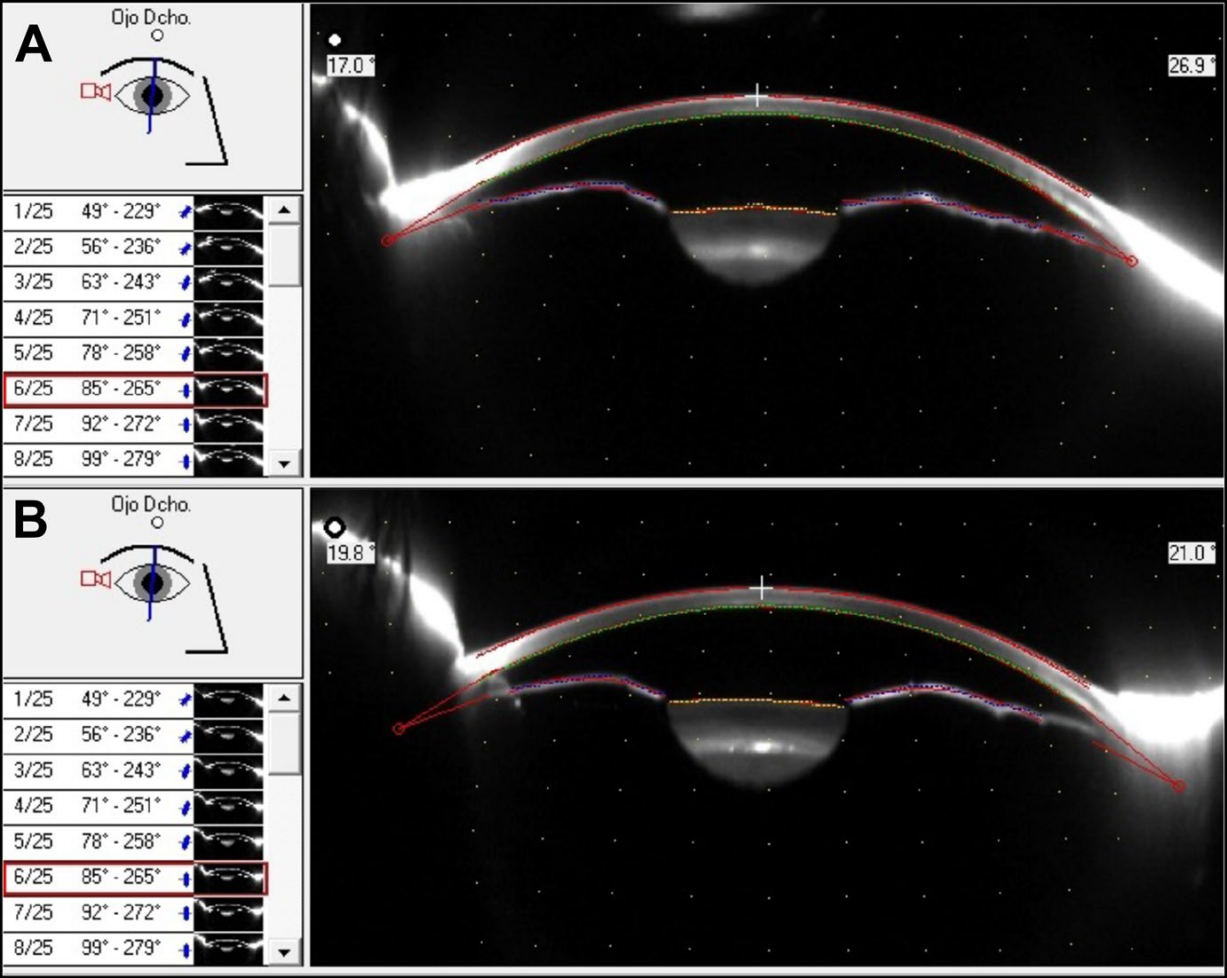
Scheimpflug imagen from Pentacam^®^ showing the decrease in the iridocorneal angle before (**A**) and 4 weeks after botulinum toxin treatment (**B**) in right eye

The anterior chamber angle decreased from 35 ± 10º to 33.8 ± 9.7º after the injection (*p* = *0.022;* Fig. 1*).* However, the mean anterior chamber depth and volume did not change significantly after botulinum toxin injection (*p* = *0.122* and *p* = *0.84*, respectively).

## Discussion

The cornea is a tissue with viscoelastic properties that give it the ability to mold. The cornea is covered by the eyelid, partially when the eye is open and completely during blinking. Thus, the eyelid tone and function may affect it. Changes in the refractive power of the cornea resulting from eyelid pressure have been reported after implantation of gold weight implants in the upper eyelid [19], congenital blepharoptosis [20], eyelid massage [21], vernal keratoconjunctivitis[22] or eyelid tumors.

Periocular botulinum toxin injection has also been shown to influence corneal parameters. Osaki et al. found a flattening of the steep axis over time after botulinum toxin injection in patients with hemifacial spasms using conventional corneal topography [23]. Moon et al., using Keratograph^®^, analyzed vectors of astigmatism changes in patients with blepharospasm and hemifacial spasm after botulinum toxin injection and showed an astigmatism change to against-the-rule astigmatism from the first month after treatment, thus documenting anterior corneal surface remodeling [24]. Zhang et al., studied keratometric differences between healthy eyes and eyes with different degrees of blepharospasm using Pentacam^®^ [25] and concluded that posterior corneal surface remodeling only occurs in cases of the moderate–severe degree of blepharospasm. However, in our study, we have not observed any differences in keratometry parameters nor in the AS or in the PS of the cornea after botulinum toxin injection. Ulusoy et al. have reported thinner central corneal thickness in patients with hemifacial spasms when compared to control eyes. This finding has been attributed to corneal hypoxia leading to stromal remodeling in eyes with hemifacial spasm [26]. In our study, we observed that botulinum toxin treatment increased slightly the central corneal thickness, however, this increase did not reach statistical significance. We interpret this slight increase in central corneal thickness could be the result of improved corneal oxygenation after botulinum toxin injection.

In this study, we find a significant decrease in the iridocorneal angle following botulinum toxin injection. Although previous studies have documented acute angle-closure glaucoma episodes after periocular botulinum toxin injection in patients with blepharospasm [14–17], this is the first study that documents narrowing of the iridocorneal angle. Periocular toxin injection paralyzes the orbicularis muscle causing decreasing blinking. How does the toxin decrease the angle is a matter of speculation. One of the most frequent side effects of periocular botulinum toxin injection is ptosis, which is believed to be secondary to diffusion of the toxin to the upper elevator muscle of the eyelid. Other frequent side effect is diplopia when the toxin reaches the oculomotor muscles [13]. Diffusion of the botulinum toxin to the ciliary ganglion, although not documented, may inhibit the cholinergic post-ganglionic neurotransmission to the ciliary muscle and the pupillary sphincter muscles, and result in mydriasis and acute angle-closure [27, 28]. Another possibility to explain the acute angle-closure [27, 28] is that the drug passes directly from the periocular tissues to the anterior chamber affecting directly the pupillary sphincter.

Since after the botulinum toxin injection we find significant differences in the anterior chamber angle but not in the keratometric values and the anterior chamber volume and depth, we interpret the reduction of the iridocorneal angle may be due not to the relaxation of the orbicularis oculi muscle but to relaxation of the pupillary sphincter. However, a limitation of the current study is that we did not measure the pupil diameter.

Periocular botulinum toxin injection should be thus used with caution in patients with narrow angles or angle-closure glaucoma because it decreases the iridocorneal angle. If applied in these patients, the intraocular pressure should be monitored closely after the procedure because of a potential acute rise in intraocular pressure.

## Conclusions

Periocular injection of botulinum toxin did not affect the anterior or posterior corneal surface parameters nor the anterior chamber depth or volume, but decreased the iridocorneal angle, and this may cause acute angle-closure glaucoma.
